# Supplementary material for: Intraglandular mesenchymal stem cell treatment induces changes in the salivary proteome of irradiated patients
Source: Commun Med (Lond). 2022 Dec 10;2:160. doi: 10.1038/s43856-022-00223-3 (PMC9735277; doi:10.1038/s43856-022-00223-3)
Supplement: Supplementary file 5 — Supplementary Information File [file 43856_2022_223_MOESM5_ESM.pdf]

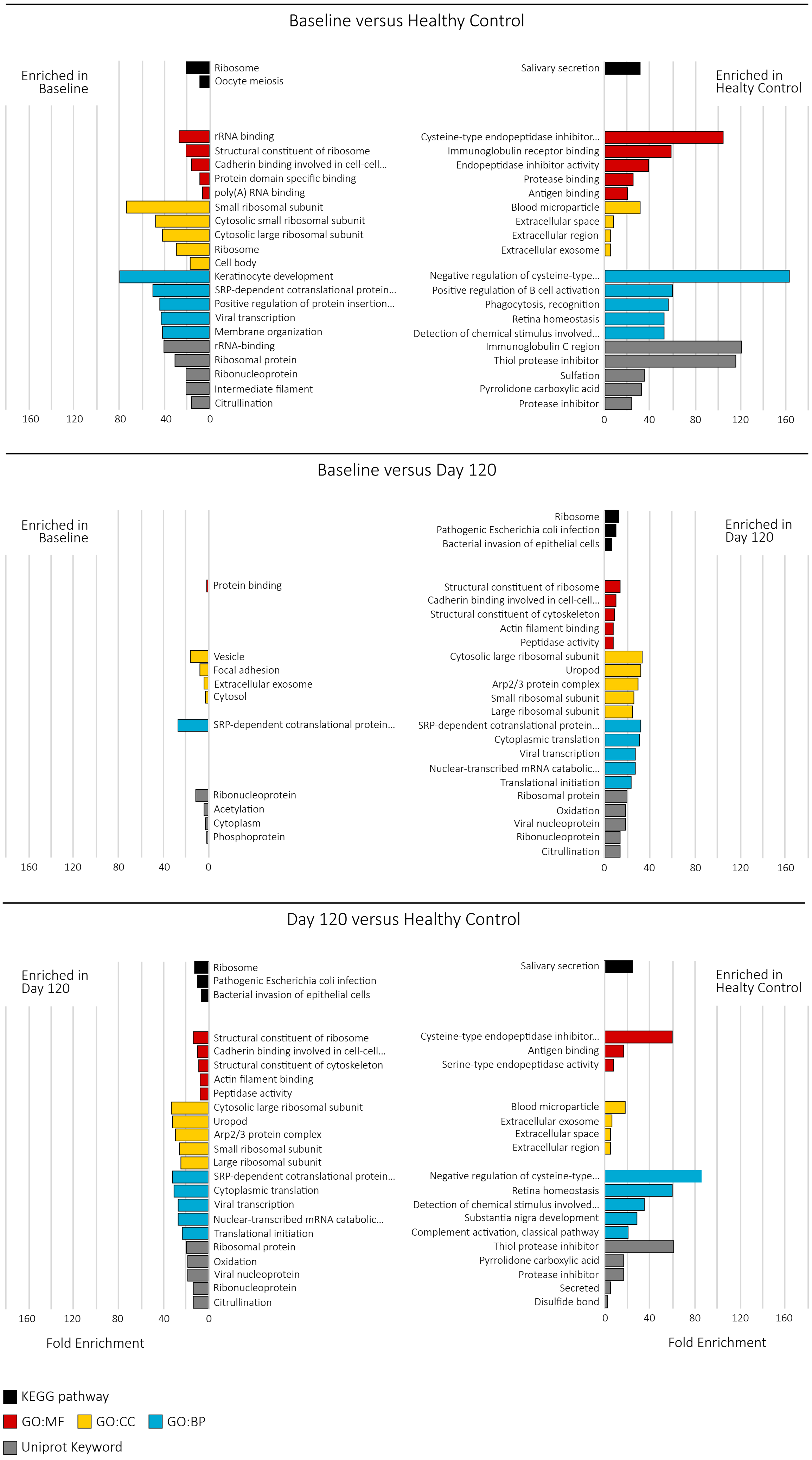

Baseline versus Healthy Control
Enriched in Baseline
Enriched in Healty Control
Ribosome
Oocyte meiosis
rRNA binding
Structural constituent of ribosome
Cadherin binding involved in cell-cell...
Protein domain specific binding
poly(A) RNA binding
Small ribosomal subunit
Cytosolic small ribosomal subunit
Cytosolic large ribosomal subunit
Ribosome
Cell body
Keratinocyte development
SRP-dependent cotranslational protein...
Positive regulation of protein insertion...
Viral transcription
Membrane organization
rRNA-binding
Ribosomal protein
Ribonucleoprotein
Intermediate filament
Citrullination
Salivary secretion
Cysteine-type endopeptidase inhibitor...
Immunoglobulin receptor binding
Endopeptidase inhibitor activity
Protease binding
Antigen binding
Blood microparticle
Extracellular space
Extracellular region
Extracellular exosome
Negative regulation of cysteine-type...
Positive regulation of B cell activation
Phagocytosis, recognition
Retina homeostasis
Detection of chemical stimulus involved...
Immunoglobulin C region
Thiol protease inhibitor
Sulfation
Pyrrolidone carboxylic acid
Protease inhibitor
160 120 80 40 0
0 40 80 120 160
Baseline versus Day 120
Enriched in Baseline
Enriched in Day 120
Protein binding
Vesicle
Focal adhesion
Extracellular exosome
Cytosol
SRP-dependent cotranslational protein...
Ribonucleoprotein
Acetylation
Cytoplasm
Phosphoprotein
Ribosome
Pathogenic Escherichia coli infection
Bacterial invasion of epithelial cells
Structural constituent of ribosome
Cadherin binding involved in cell-cell...
Structural constituent of cytoskeleton
Actin filament binding
Peptidase activity
Cytosolic large ribosomal subunit
Uropod
Arp2/3 protein complex
Small ribosomal subunit
Large ribosomal subunit
SRP-dependent cotranslational protein...
Cytoplasmic translation
Viral transcription
Nuclear-transcribed mRNA catabolic...
Translational initiation
Ribosomal protein
Oxidation
Viral nucleoprotein
Ribonucleoprotein
Citrullination
160 120 80 40 0
0 40 80 120 160
Day 120 versus Healthy Control
Enriched in Day 120
Enriched in Healty Control
Ribosome
Pathogenic Escherichia coli infection
Bacterial invasion of epithelial cells
Structural constituent of ribosome
Cadherin binding involved in cell-cell...
Structural constituent of cytoskeleton
Actin filament binding
Peptidase activity
Cytosolic large ribosomal subunit
Uropod
Arp2/3 protein complex
Small ribosomal subunit
Large ribosomal subunit
SRP-dependent cotranslational protein...
Cytoplasmic translation
Viral transcription
Nuclear-transcribed mRNA catabolic...
Translational initiation
Ribosomal protein
Oxidation
Viral nucleoprotein
Ribonucleoprotein
Citrullination
Salivary secretion
Cysteine-type endopeptidase inhibitor...
Antigen binding
Serine-type endopeptidase activity
Blood microparticle
Extracellular exosome
Extracellular space
Extracellular region
Negative regulation of cysteine-type...
Retina homeostasis
Detection of chemical stimulus involved...
Substantia nigra development
Complement activation, classical pathway
Thiol protease inhibitor
Pyrrolidone carboxylic acid
Protease inhibitor
Secreted
Disulfide bond
160 120 80 40 0
0 40 80 120 160
Fold Enrichment
Fold Enrichment
KEGG pathway
GO:MF
GO:CC
GO:BP
Uniprot Keyword

Supplementary figure 2

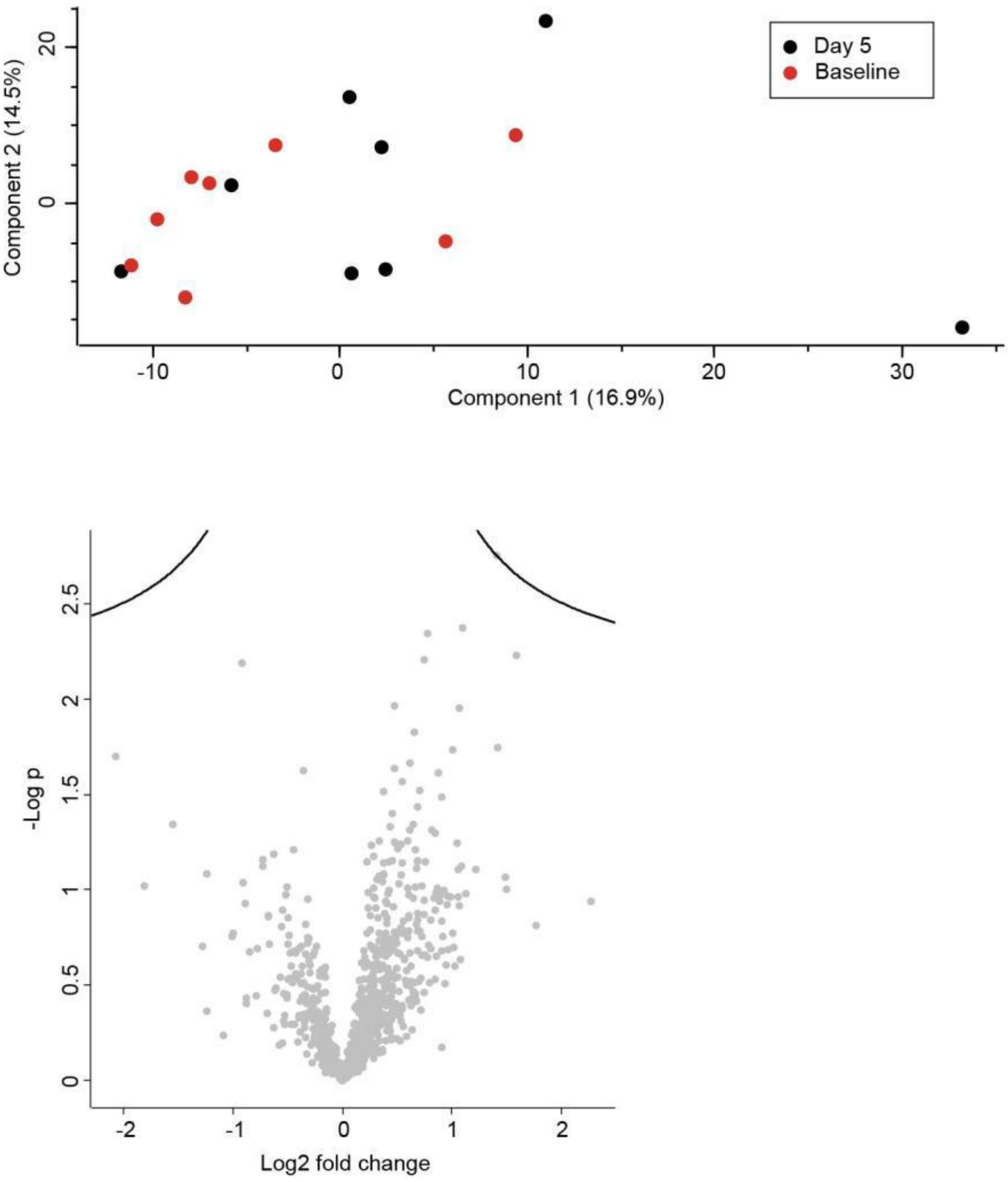

Supplementary Figure 1; Comparison of baseline and day 5. A) Principal Component Analysis (PCA) plot showing no clear separation of groups. B) Volcano plot showing no significantly differentially expressed proteins between groups.
