# Supplementary material for: Intraglandular mesenchymal stem cell treatment induces changes in the salivary proteome of irradiated patients
Source: Commun Med (Lond). 2022 Dec 10;2:160. doi: 10.1038/s43856-022-00223-3 (PMC9735277; doi:10.1038/s43856-022-00223-3)
Supplement: Supplementary file 6 — Description of Additional Supplementary Files [file 43856_2022_223_MOESM6_ESM.pdf]

Description of Additional Supplementary Files

File Name: Supplementary Data 1

Description: Baseline salivary proteome from patients treated with radiotherapy compared to healthy controls

File Name: Supplementary Data 2

Description: Baseline salivary proteome from patients treated with radiotherapy compared to salivary proteome 120 days after AT-MSC therapy

File Name: Supplementary Data 3

Description: Salivary proteome 120 days after AT-MSC therapy compared to healthy controls

File Name: Supplementary Data 4

Description: Data for Fig. 2-6.
