# Supplementary material for: Intraglandular mesenchymal stem cell treatment induces changes in the salivary proteome of irradiated patients
Source: Commun Med (Lond). 2022 Dec 10;2:160. doi: 10.1038/s43856-022-00223-3 (PMC9735277; doi:10.1038/s43856-022-00223-3)
Supplement: Supplementary file 8 — Reporting Summary [file 43856_2022_223_MOESM8_ESM.pdf]

Corresponding author(s): Charlotte Duch Lynggaard

Last updated by author(s): Nov 13, 2022

Reporting Summary

Nature Research wishes to improve the reproducibility of the work that we publish. This form provides structure for consistency and transparency in reporting. For further information on Nature Research policies, see our Editorial Policies and the Editorial Policy Checklist.

Statistics

For all statistical analyses, confirm that the following items are present in the figure legend, table legend, main text, or Methods section.

n/a	Confirmed	
☐	☒	The exact sample size (*n*) for each experimental group/condition, given as a discrete number and unit of measurement
☐	☒	A statement on whether measurements were taken from distinct samples or whether the same sample was measured repeatedly
☐	☒	The statistical test(s) used AND whether they are one- or two-sided<br>*Only common tests should be described solely by name; describe more complex techniques in the Methods section.*
☐	☒	A description of all covariates tested
☐	☒	A description of any assumptions or corrections, such as tests of normality and adjustment for multiple comparisons
☐	☒	A full description of the statistical parameters including central tendency (e.g. means) or other basic estimates (e.g. regression coefficient) AND variation (e.g. standard deviation) or associated estimates of uncertainty (e.g. confidence intervals)
☒	☐	For null hypothesis testing, the test statistic (e.g. *F*, *t*, *r*) with confidence intervals, effect sizes, degrees of freedom and *P* value noted<br>*Give P values as exact values whenever suitable.*
☒	☐	For Bayesian analysis, information on the choice of priors and Markov chain Monte Carlo settings
☐	☒	For hierarchical and complex designs, identification of the appropriate level for tests and full reporting of outcomes
☐	☒	Estimates of effect sizes (e.g. Cohen's *d*, Pearson's *r*), indicating how they were calculated

Our web collection on statistics for biologists contains articles on many of the points above.

Software and code

Policy information about availability of computer code

Data collection	Raw data were processed with Proteome Discoverer version 2.4
Data analysis	Output from Proteome Discoverer version 2.4 was analyzed with Perseus version 1.6.5.0

For manuscripts utilizing custom algorithms or software that are central to the research but not yet described in published literature, software must be made available to editors and reviewers. We strongly encourage code deposition in a community repository (e.g. GitHub). See the Nature Research guidelines for submitting code & software for further information.

Data

Policy information about availability of data

All manuscripts must include a data availability statement. This statement should provide the following information, where applicable:

- Accession codes, unique identifiers, or web links for publicly available datasets
- A list of figures that have associated raw data
- A description of any restrictions on data availability

The mass spectrometry proteomics data have been deposited to the ProteomeXchange Consortium via the PRIDE partner repository with the dataset identifier PXD024152. All other data supporting the findings of this study are available from the corresponding authors upon reasonable request. Source data for the figures available as Supplementary Data for figures.

Field-specific reporting

Please select the one below that is the best fit for your research. If you are not sure, read the appropriate sections before making your selection.

☒ Life sciences ☐ Behavioural & social sciences ☐ Ecological, evolutionary & environmental sciences

For a reference copy of the document with all sections, see nature.com/documents/nr-reporting-summary-flat.pdf

Life sciences study design

All studies must disclose on these points even when the disclosure is negative.

Sample size	The study was an explorative extension of an investigator-initiated, first-in-human, non-randomized, open-label, phase I clinical trial evaluate the safety and efficacy of using allogeneic AT-MSCs to treat radiation-induced hyposalivation and designed to include ten patients. We planned to perform the analysis of all the included patients, but only eights produced an adequate amount of saliva (1mL) for the analysis.
Data exclusions	No data were excluded.
Replication	No replication tests were performed.
Randomization	The patients were not randomized.
Blinding	The study was not blinded.

Reporting for specific materials, systems and methods

We require information from authors about some types of materials, experimental systems and methods used in many studies. Here, indicate whether each material, system or method listed is relevant to your study. If you are not sure if a list item applies to your research, read the appropriate section before selecting a response.

Materials & experimental systems

n/a	Involved in the study	
☒	☐	Antibodies
☒	☐	Eukaryotic cell lines
☒	☐	Palaeontology and archaeology
☒	☐	Animals and other organisms
☐	☒	Human research participants
☐	☒	Clinical data
☒	☐	Dual use research of concern

Methods

n/a	Involved in the study	
☒	☐	ChIP-seq
☒	☐	Flow cytometry
☒	☐	MRI-based neuroimaging

Human research participants

Policy information about studies involving human research participants

Population characteristics	Main inclusion criteria comprised patients of both sexes between 18-75 years, with previous OPSCC stage I-II (The Union for International Cancer Control 8th, UICC 8), with symptoms and objective measures of radiation damage of the SGs with a minimum of 2 years without relapse after radiation therapy. Main exclusion criteria were cancer in the previous 4 years (not including OPSCC and basal cell carcinomas), xerogenic medications, penicillin or streptomycin allergy, other diseases of the SGs, any previous stem cell therapy or SG surgery, alcohol abuse, smoking, or pregnancy (A full list of eligibility criteria is registred on ClinicalTrials.gov, number NCT03874572). Patients were screened for human immunodeficiency virus (HIV),
Recruitment	Patients were recruited from the Department of Otolaryngology, Head and Neck Surgery and Audiology at Rigshospitalet, the Departments of Oncology at Rigshospitalet and Herlev-Gentofte Hospital, or through self-referral triggered by media awareness.
Ethics oversight	The trial was conducted according to the original protocol and complied with the Declaration of Helsinki and was approval obtained from the National Committee on Health Research Ethics (H-1808924) and the Danish Medicines Agency (Eudra-CT 2018-003856-19). The trial was monitored by the Good Clinical Practice (GCP) Unit of Copenhagen.

Note that full information on the approval of the study protocol must also be provided in the manuscript.

Clinical data

Policy information about clinical studies

All manuscripts should comply with the ICMJE guidelines for publication of clinical research and a completed CONSORT checklist must be included with all submissions.

Clinical trial registration	The trial protocol was registered at ClinicalTrials.gov (number NCT03874572)
Study protocol	The trial protocol is published in Stem Cells Translational Medicine
Data collection	Dr. Charlotte Duch Lynggaard performed collection of the saliva. Dr. Rosa Jersie-Christensen performed the analysis.
Outcomes	We evaluated possible changes in the salivary proteome in samples from day 0 (intervention), day 5 and day 120.
